# Italian Adolescent Young Caregivers of Grandparents: Difficulties Experienced and Support Needed in Intergenerational Caregiving—Qualitative Findings from a European Union Funded Project

**DOI:** 10.3390/ijerph19010103

**Published:** 2021-12-23

**Authors:** Barbara D’Amen, Marco Socci, Mirko Di Rosa, Giulia Casu, Licia Boccaletti, Elizabeth Hanson, Sara Santini

**Affiliations:** 1 Centre for Socio-Economic Research on Aging, IRCCS INRCA-National Institute of Health and Science on Aging, Via Santa Margherita 5, 60124 Ancona, Italy; b.damen@inrca.it (B.D.); s.santini2@inrca.it (S.S.); 2 Laboratory of Geriatric Pharmacoepidemiology, IRCCS INRCA-National Institute of Health and Science on Aging, Via Santa Margherita 5, 60124 Ancona, Italy; m.dirosa@inrca.it; 3 Department of Psychology “Renzo Canestrari”, University of Bologna, Viale Berti Pichat 5, 40127 Bologna, Italy; giulia.casu3@unibo.it; 4 Anziani e Non Solo Società Cooperativa Sociale, Via Lenin 55, 41012 Carpi, Italy; progetti@anzianienonsolo.it; 5 Department Health and Caring Sciences, Linnaeus University, SE-39182 Kalmar, Sweden; elizabeth.hanson@lnu.se; 6 The Swedish Family Care Competence Centre, Strömgatan 13, SE-39232 Kalmar, Sweden

**Keywords:** adolescent young caregivers (AYCs), caregiving difficulties, support needed, grandparents (GrPs), descriptive study

## Abstract

The article aims to describe the experiences of 87 Italian adolescent young caregivers (AYCs) of grandparents (GrPs), with reference to the caregiving stress appraisal model (CSA) that provides a theoretical lens to explore the difficulties encountered and support needed in their caring role. Qualitative data were drawn from an online survey conducted within an EU Horizon 2020 funded project. An inductive thematic analysis was carried out, and the findings were critically interpreted within the conceptual framework of the CSA model. The analysis highlighted three categories of difficulties: material, communication and emotional/psychological. The most common material difficulty was the physical strain associated with moving “uncooperative” disabled older adults. The types of support needed concerned both emotional and material support. The study provides a deeper understanding of the under-studied experiences of AYCs of GrPs. Based on these findings, policies and support measures targeted at AYCs of GrPs should include early needs detection, emotional support and training on intergenerational caring in order to mitigate the stress drivers. Moreover, the study advances the conceptualisation of the CSA model by considering the above-mentioned aspects related to intergenerational caregiving.

## 1. Introduction

Adolescent young caregivers (AYCs) of grandparents (GrPs) are youngsters in the middle phase of adolescence, e.g., between 15 and 17 years, who carry out significant or substantial caring responsibilities for an older, sick or disabled relative [1]. AYCs of GrPs remain an under-researched group of informal caregivers in Italy as well as in Europe [2]. However, the phenomenon of adolescent and young people taking care of a family member is more and more widespread due to several factors, e.g., changes in the labour market (the growing number of working caregivers) and in the family organisation and composition (geographic mobility and single-parent families) [3]. Moreover, an ageing society [4] and the corresponding increase in long-term care demand questioning both the formal and informal care sector [5] are further drivers for involving young people in the assistance of (older) family members.

To the best of our knowledge, there are no precise figures about AYCs in general nor about AYCs of GrPs across Europe today. Nevertheless, national statistics about young caregivers in different European countries suggest that about 7–8% of children in Europe have caregiving responsibilities [6]. Concerning Italy, the second wave of the European Health Interview Survey highlighted that 6.6% of Italian youngsters aged from 15 to 24 years provide care to a family member [7]. Despite these data, the empirical literature focusing on AYCs is limited. Further, there is a lack of consistency regarding the definition of AYCs [8,9,10] across the available studies. Moreover, carrying out research on AYCs is challenging especially because of young people’s low level of self-recognition that makes it difficult for them to be identified and to be enrolled in projects and interventions [11,12].

The paucity of knowledge around this topic is one of the reasons for the general lack of awareness, visibility and support of AYCs across Europe, with the exception of the United Kingdom (UK), which has approximately 30 years of research on this topic and overall a greater awareness [13] and a variety of laws (even without systematic legislation) [9]. 

The available literature on AYCs shows that AYCs can be engaged to different extents along the continuum of care, also depending on the available formal and informal support services, from caring about to caring for an (older) family member [14]. In this regard, the few previous studies focusing on AYCs of GrPs revealed that they often play the role of the auxiliary caregiver [15], helping their older relatives with instrumental activities of daily living (IADLs) [16], and providing companionship and emotional support [17]. AYCs of GrPs can experience many types of emotional difficulties while providing care, such as fear of what might happen to their GrPs due to their illness (which they often do not know exactly) and feelings of guilt if they do not provide care. At the same time, AYCs may experience both positive and negative caregiving outcomes. Positive outcomes include greater resilience [18], maturity [19], empathy [20] and an enhanced self-image [21]. Whilst negative outcomes include mental health problems, poor well-being [22,23,24], stress [25] and social isolation [26]. 

To the best of our knowledge, few studies shed light on the conditions of AYCs of GrPs by framing them within a conceptual framework, and there are no prior studies that have focused on Italian AYCs [2]. In light of the above, this study has two objectives. The first is to advance the knowledge about intergenerational caregiving by reporting on the qualitative findings concerning a group of Italian AYCs of GrPs involved in an online survey within the context of the EU-funded Me–We project (see below). 

The second objective is to create a more in-depth understanding of the phenomenon by making use of the main empirical findings to put forward the essence of an evidence-based conceptual framework on AYCs of GrPs. To this purpose, the findings were framed within the caregiving stress appraisal model (renamed CSA model) proposed by Yates et al. [27]. 

### The Italian Context: Long Term Care (LTC) System and Legislation on Caring

The progressive population aging [28] and increasing demand for long-term care (LTC) [29], especially in countries with a less well-developed LTC system, such as Italy [30], may drive the involvement of minors in caring for an older family member to counteract the dearth of formal support. The Italian context is characterised by an LTC extensively based on cash-for-care measures, with limited home care in-kind services [31], and, in general, to the extent that is not sufficient for addressing the need of care of the growing older population with multiple chronic diseases [30]. In addition, it is possible that the family-oriented welfare system operating in Italy [32,33] together with several changes in the labour market and in family settings, e.g., an increasing number of employed women, lack of strong family networks, living in single parent families [34], can turn young people into family caregivers.

According to Leu and Becker’s classification system of country awareness and policy responses to AYCs [35], Italy has been categorised as an “emergent” country that means a paucity of services, research and AYCs’ legal recognition. Indeed, in Italy, there is a lack of specific national legislation on AYCs. In fact, a draft national law “for the recognition and support of the family caregiver” [36] is still pending in the Italian Senate. 

Nevertheless, some Italian regions, e.g., Abruzzo, Campania and Emilia Romagna, adopted laws that apply in their territories for relief and support interventions for family caregivers [37,38]. In these regions, several support services were implemented for family caregivers, and the few psychological or socio-educational interventions addressed to the youngsters remain limited to particular territories and are not widespread. Overall, the lack of specific laws and, at the same time, the lack of adequate development of support services in Italy, often exacerbated by the absence of funding, makes AYCs more exposed to difficulties and, in general, unable to find adequate responses to their support needs. 

## 2. Materials and Methods

### 2.1. Procedure and Study Design

The online survey was carried out between March and October 2018 in six European countries (Italy, the Netherlands, Slovenia, Sweden, Switzerland and the UK) within the “Psychosocial support for promoting mental health and well-being among adolescent young caregivers in Europe” (Me–We) EU Horizon 2020 research and innovation project (2018–2021, https://me-we.eu, accessed on 20 June 2021). 

It was based on a questionnaire aimed at profiling AYCs by collecting quantitative and qualitative data. As regards the quantitative data, the questionnaire was comprised of a demographic section with questions about age, gender, nationality, living conditions, etc. In order to evaluate the positive and negative effects of caregiving activity, the “Positive and Negative Outcomes of Caring” (PANOC-YC20), a 20-item self-report measure was utilised [39]. The impact of caregiving on the youngsters’ health was measured by means of “KIDSCREEN-10”, a 10-item measure of the health-related quality of life standard, in which each item is answered on a 5-point frequency response scale, ranging from 10 to 50, where higher values indicate the better health-related quality of life [40,41]. Moreover, an ad hoc developed single-item measure was added, named “Health problems in connection with caring” [42].

The qualitative data were collected through two open-ended questions. The first one was focused on the help that would support the respondents as a carer, whilst the second was focused on the main difficulties encountered by AYCs in providing assistance to GrPs. 

The data analysis was based on a simultaneous mixed-method design [43] comprising a qualitatively-driven core component and a quantitative supplementary component. Thus, the quantitative data are not related to the qualitative ones; they are rather used purely for presenting the characteristics of the study participants.

**Figure 1:**
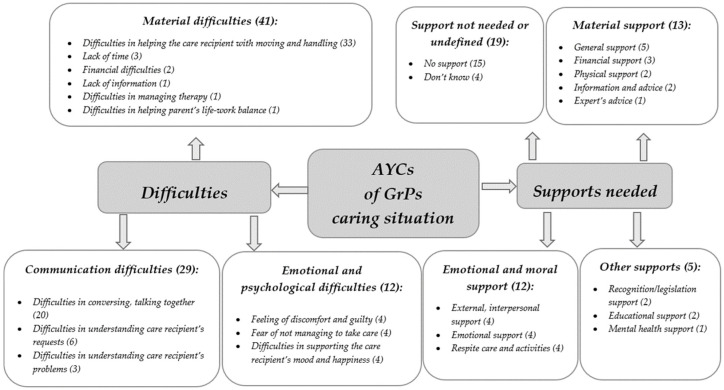
Themes and subthemes related to difficulties and support needs of AYCs of GrPs.

In light of this study design, the qualitative responses related to the above-mentioned questions were selected and analysed by applying an open coding process [44], a method through which concepts and their dimensions are identified and discovered directly from the data. Following the open coding, codes referring to the same phenomenon were grouped into sub-themes, and these were subsequently grouped into higher-order themes. The analysis was conducted with the support of MAXQDA 2020 software (VERBI Software, Berlin, Germany) and highlighted major themes, organised into sub-themes that finally were described in conceptual maps [45], in order to provide a clear description of the findings (Figure 1 in the Results section). 

The latter were framed within the CSA model [27]. This model, derived from both the stress model presented by Pearlin et al. [46] and the appraisal model of Lawton et al. [47,48], links caregiving stressors, caregiving appraisal and potential mediators to caregiver well-being. According to this model, caregiving is considered as a complex process in which, in addition to variables of care recipient’s needs for care, named “primary stressors” (i.e., cognitive impairments, disability, etc.), two separate caregiver appraisals affect the relationship between the stressors and the outcomes: the primary and secondary appraisal. The primary appraisal (i.e., hours of informal care, which is the response to the care recipient’s health conditions) includes both subjective elements (e.g., appraisal of the care recipient’s needs of care) as well as objective ones (e.g., the measure of caregiving work). 

The secondary appraisal is related to the perception of being “overloaded”, which is the caregiver’s capability of determining their own feelings about caring. In this process, specific mediators could change the effects of the stressor on the caregiver’s well-being: these are classified as external (e.g., use of formal services) and internal (e.g., levels of global mastery, quality of the relationship between the caregiver and the care recipient, and emotional support available to the caregiver). Hence, the CSA model defines the caregiving outcomes, i.e., the caregiver’s psychological well-being, measured by the risk of depression, and highlights the association between the caregiver’s overload and consequent depression. 

### 2.2. Study Participants

A convenience sample of 817 European AYCs aged 15–17 were surveyed through an online questionnaire aimed to guarantee participants’ anonymity and privacy on different types of electronic devices, e.g., personal computers and smartphones. In Italy, respondents were mainly recruited among high school students from a region in the north and a region in the centre of Italy, respectively. Ethics approval was obtained from the Ethics Board of the University “Alma Mater Studiorum” of Bologna (Italy), Department of Psychology, on 21 December 2017. 

By filling in the questionnaire, respondents gave their informed consent to take part in the study. An information letter and the first page of the online questionnaire made it clear that AYCs’ participation was voluntary and that they could withdraw at any time without any explanation. Informed consent was also secured from parents/legal guardians in accordance with applicable national legislation and institutional guidance. The data were processed in full compliance with both national laws on data protection and the General Data Protection and Regulation (EU 2016/679; Regulation, G.D.P.R., 2016) [49].

Out of the 817 respondents, 264 (32%) provided care for older adults, and 87 (33%) were Italian.

## 3. Results

### 3.1. Sample Characteristics

The 87 surveyed Italian AYCs (Table 1) were mainly female (66.7%) and born in Italy (93.1%). Most of them were 16 and 17 years old (40.2% and 48.3%, respectively), and the average age at which they started providing care was 12.8 years. Care recipients (their GrPs, i.e., older adults aged 65 years and over) suffered mainly from physical disabilities (62.1%) or cognitive impairments (35.6%). The AYCs of GrPs reported caring 2.7 h a day on average, and their family received mostly informal support (namely, help from wider family members and/or friends) (33.3%). With regards to the personal impact of their caring role, few AYCs reported health problems (32.2%), whilst they reported significantly higher positive outcomes (e.g., feeling that “I’m helping, closer to family”) than negative outcomes (e.g., feeling stressed or lonely) in the PANOC-YC20 and an average score related to the KIDSCREEN that confirms an overall positive quality of life. 

### 3.2. Qualitative Data Analysis

#### 3.2.1. Difficulties Encountered by AYCs of GrPs

Considering the difficulties encountered by AYCs of GrPs, a qualitative analysis allowed the project researchers to elucidate three core themes, i.e., “material”, “communication”, and “emotional and psychological difficulties” (Figure 1).

“Material difficulties” are the most common ones experienced by AYCs and were reported in 41 quotations. The main subtheme concerns “difficulties in helping the care recipient with moving and handling”, reported by 33 respondents and directly connected with the support that AYCs provide in relation to GrPs’ activities of daily living (ADLs) [16]. Moreover, this subtheme is also connected with the relationship between AYCs and GrPs, showing the physical burden as a consequence of the age/weight gap. An example of this aspect/theme/subtheme is clarified by the following quotation:

*“Her body weight is heavy for me and the smell of certain leakages is disgusting”*

Other subthemes that emerged from eight quotations are mostly related to external aspects of the caregiving relationship, such as organisational ones. These include “lack of time” for providing caregiving tasks, “financial difficulties”, “lack of information” on caring, difficulties in managing the care recipient’s therapy and in helping parents’ life-work balance. The latter suggests that the AYCs can often be required to provide double support, not only to older adults but also to their own parents. 

The second theme concerns “communication difficulties”, reported by 29 young people. In particular, most of them (20) reported “difficulties in conversing and talking together” with the care recipient/s, whilst nine respondents reported difficulties in understanding the care recipient’s requests and problems. These subthemes are closely connected with the caregiving relationship, the age gap and the care recipient’s illness (e.g., cognitive impairments or hearing difficulties), as depicted by the following quotation.

*“I find difficult to understand my grandma: she has speech and hearing difficulties”*

The “emotional and psychological difficulties” reported by 12 respondents underline AYCs’ discomfort as a consequence of the above-mentioned factors, such as the care recipient’s illness and age gap. As shown in Figure 1, the identified subthemes include “feeling of discomfort and guilt” (reported by four respondents) due to the direct contact with the care recipient’s illness, and also possibly feelings of helplessness in being able to support them.

*“I simply feel deeply sad to see my paternal grandparents in the state they are, suffering from dementia”*

Furthermore, the sense of guilt seems to be a consequence of the frustration of AYCs towards the difficulties faced in managing their care recipient’s illness/es.

*“Being able to endure a person who constantly repeats the same things to exhaustion, and be able to bear the sense of guilt you feel when you answer them wrong by blaming them”*

The second subtheme, reported by four respondents, is the “fear of not managing to take care” of their GrPs that could lead AYCs to a sense of inadequacy and helplessness.

*“I’m afraid something serious could happen in my presence, for example once my grandfather had an epileptic crisis in front of me, I was traumatized from fear and I called for the ambulance”*

The last subtheme is the “difficulty in supporting the care recipient’s mood and happiness” reported by four respondents.

*“The main difficulty is to make my grandma smile really”*

#### 3.2.2. Type of Supports Needed by AYCs of GrPs

The question “If you are looking after someone, what would help support you as a carer?” was answered by 49 AYCs of GrPs. Fifteen respondents stated they did not currently receive any support. Among these, five youngsters reported this as a consequence of not being in the “frontline” of care but helping their parents in their role of primary caregivers. Two respondents stated that their willingness to take on the caregiving role was due to family bonds, and as a result, they did not feel the need for any kind of support. Eight respondents did not specify the reason why they did not receive any support, while four declared not to know. 

In line with the evidence related to the difficulties encountered by AYCs, the second theme arising from the analysis is the need for “material support” (13 respondents). Concerning this, the most common subtheme concerns “general support” (five respondents), referral to a person that could provide generic help. Only three respondents declared the need for “financial support”, which could also be a way of recognising AYC’s efforts, as clarified in the following quotation:

*“I need financial support from the state, since the merits and above all the great sacrifice of a boy who puts the lives of others first in relation to his must be recognized.”*

Other subthemes relate to the need for “physical support” (two respondents), “information and advice” (two respondents), whilst only one respondent declared the need for “experts’ support” (e.g., a doctor), as depicted by the quotations below.

*“Help them with things they can’t do”*

*“I need advice since I’m not specialized in certain areas… I would need advice”*

*“In the role of caregiver, I would need a doctor or a psychologist for sure by my side so I would know how to help a person with the disease”*

A further theme is a need for “emotional and moral support” (12 respondents), which for four youngsters could be provided by a person outside of the family network, who could be available to listen to and give advice. Four respondents referred to the need for “emotional support” related to psychological and emotional skills (e.g., patience) for themselves, whilst four respondents declared the need for more “free time” and the opportunity for a “respite” from care activities.

*“Don’t be alone and have more information to be able to help more”*

*“Patience”*

*“I need more time for myself”*

Finally, two respondents reported the need for support to improve awareness of AYCs and their activities, and two other AYCs stressed the need for “educational support”. A respondent declared that dedicated mental health support services are needed. 

## 4. Discussion

This study had the twofold objective of (1) advancing the knowledge about the intergenerational caregiving relationship between a group of Italian AYCs and their GrPs for providing possible directions for future policies and supports for this understudied group, and (2) contributing to the conceptualisation of a model addressing the experiences of AYCs of GrPs. 

### 4.1. Intergenerational Caregiving and Suggestions for Policy and Support Services

In order to address the first objective, the difficulties experienced and the support needed by Italian AYCs of GrPs were examined by analysing qualitative data drawn from two open-ended questions asked in an online survey. 

The analysis highlighted three main themes: “material difficulties”, “communication difficulties” and “emotional and psychological difficulties”. Each theme provides suggestions for improving policies and services for AYCs.

Among the “material difficulties”, the “difficulties in helping the care recipient with moving and handling” seem to mirror the lack of policies and formal support services for informal caregivers in Italy. It can be argued that the AYCs of this study moved the older family member by themselves because they are not appropriately helped by formal home care services and/or devices (e.g., patient hoist) that can guarantee constant in-home assistance [50]. This points to the need for formal home care services to be increased in Italy for supporting informal caregivers in managing daily caregiving activities and preventing youngsters from being involved in moving and handling older family members. 

Moreover, AYCs of this study acknowledged in their responses that they lacked the necessary skills to move their disabled and “uncooperative” grandparents safely by adopting techniques for limiting the consequences of such efforts on their physical health, e.g., back pain, unlike care professionals who receive formal training and access to assistive devices to make moving easier and safer for them. In an ideal world, youngsters should not have the responsibility of providing care but, in the absence of alternatives, it can be argued that training should at the very least be made available for teaching AYCs how to move the care recipient safely and effectively to minimise risks to their own physical health and emotional well-being. 

Focusing on AYCs of GrPs, it is worth highlighting that several difficulties that emerged from the analysis are attributable to and exacerbated by the communication barriers connected with the poor and multi-chronic health condition of the older care recipient (e.g., problems with speech, comprehension). This suggests that providing training to AYCs of GrPs aimed at fostering an appropriate communication with older adults with sensory and/or cognitive impairment may improve intergenerational communication, reduce the AYCs’ sense of guilt and frustration [51] that may lead to “emotional and psychological difficulties” and hinder the association of the GrPs’s disease with a negative and stereotyped view of ageing [17]. 

Most of the respondents provided generic answers on the supports needed or did not realise that they needed help. Few respondents could identify what kind of support they really needed—in particular, with regards to material support—and neither could they identify the person/professional they could turn to for help. These findings mirror the low level of self-awareness of AYCs in Italy, similarly to many other countries [11,12], and indicate the need for education and awareness-raising interventions to be carried out in schools and in other locations where young people congregate. In several cases, the willingness to care for GrPs driven by emotional bonds buffered the respondents’ demand for support [52]. This highlights the importance of educational interventions and support measures adapted to the specific relationship between AYCs and GrPs, which is often characterised by affection and by the willingness of AYCs to reciprocate the care received by grandparents during their childhood.

The need for advice confirms the urgency of training for AYCs that should be tailored to the specific health condition/s of the care recipient. In this regard, when the latter is an older adult, healthcare professionals (e.g., nurses and psychologists) should be trained to design and teach educational materials for AYCs of GrPs focused on age-related diseases such as Parkinson’s and dementia. Such materials should be aimed at better understanding the multidimensional needs of older adults with long-term health issues. Moreover, healthcare professionals should be trained to provide psychological and emotional support to AYCs for improving their resilience and mental health.

The need for “free time” and “leisure” expressed by several AYCs confirms that respite services for informal caregivers are currently lacking in Italy. If respite care services are important for adult caregivers, it can be argued that they are likely to be equally important for AYCs. In fact, even if a reasonable amount of caring about an older family member may help develop AYCs’ maturity, compassion and empathy [19,20,21], it can also be argued that they also need some free time for socialisation and sport, experiences that may help build their personality, help them to flourish and transition smoothly into adulthood. 

In light of the expressed need for “emotional support”, it can be argued that the promotion of support groups for AYCs of GrPs could be a desirable source of support [51] that could be established within the educational system and/or offered through existing social service providers [2,25].

The recognition of AYCs’ needs may prevent AYCs’ stress and, consequently, promote their health and well-being. It is therefore recommended that teachers are trained to identify young caregivers among their students and to contact social services for planning tailored support measures.

Thus, specific policies and services tailored to AYCs’ needs and with due attention to the (older) care recipient’s health condition, should be designed in cooperation with both parties and, preferably together with adult caregivers living in the same household, in accordance with a family-centered approach [8]. 

### 4.2. Towards the Conceptualisation of a Caregiving Model Mirroring the Experience of AYCs’of GrPs 

**Figure 2:**
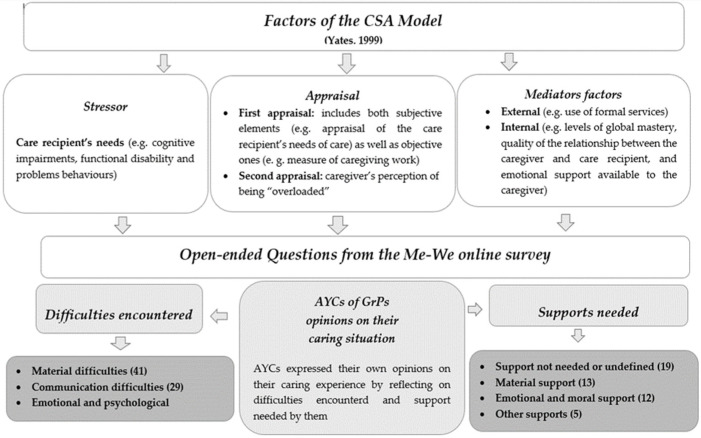
The CSA model integrated with study findings.

In order to provide insights for a theoretical understanding of AYCs of GrPs, the discussion will now focus on the delineation of a proposed conceptual framework building on the qualitative findings from the Me–We online survey study. The themes and subthemes identified through the thematic analysis were framed in the CSA model dimensions [27]. As shown in Figure 2, a. the difficulties encountered in caregiving were read as stressors, b. and answering the open-ended questions was considered as a form of appraisal, since this action entailed self-reflection; c. the support needs helped to identify measures (mediators in the CSA model) that are able to mitigate the negative caregiving outcomes and hopefully improve AYCs’ well-being. 

**Figure 3:**
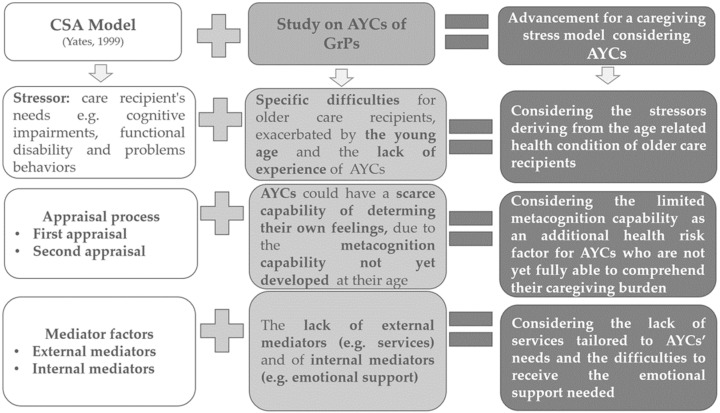
Advancement for the conceptualisation of a caregiving stress model suitable for AYCs of GrPs.

We consider that the study findings can enrich the CSA model with three evidence-based elements that should be considered in order to design a model that addresses the experiences of AYCs of GrPs. The additional elements are highlighted in Figure 3 below.

The first element is the stressor coming from the multi-chronic health condition of care recipients representing an aspect that may complicate caregiving activity because it requires multiple competencies that an adolescent young person may not yet have developed sufficiently. The second aspect to reflect on for adapting the CSA model to the experiences of AYCs of GrPs, is the appraisal process. In the CSA model, Yates assumed that caregivers are able to identify their level of overload by assessing their own situation and their feelings about caring. This perspective can be true for adult caregivers, but it may not be appropriate for AYCs because they generally have a lower self-awareness of stressors [53] compared to adults, and a low level of metacognition or introspection, especially in early adolescence [54].

Thus, the low level of metacognition that characterises adolescents could potentially worsen the emotional and psychological condition of AYCs of GrPs, leading to feelings of guilt and frustration. Similarly, the limited metacognition capability could have hindered the identification of supports needed. Therefore, a caregiving conceptual framework focusing on AYCs experiences and emotions should consider younger adolescents’ generally more limited metacognition capability as an additional health risk factor. 

Third, a conceptual framework suited to AYC’s caring situation should consider the reduced access to formal and informal support services that characterises all AYCs, and especially the sub-group caring for GrPs, due to the overall low level of self-awareness and more limited capability of identifying the typology of support needed and professionals who can provide help, as shown by this study.

### 4.3. Limitations and Suggestions for Future Research

This qualitative analysis is focused on two open-ended questions included in an online survey, answered by a sub-group of Italian AYCs of GrPs in order to explore the difficulties they encountered and the corresponding support needed. The Me–We survey is the first systematic research shedding light on AYCs across Europe, and this study is one of the first to highlight the difficulties and the support needs of a group of Italian AYCs involved in intergenerational caregiving. Nevertheless, this study has several limitations. 

First, the small sub-sample of Italian AYCs of GrPs were recruited solely in two Italian regions; thus, they cannot be considered as representative of the entire Italian situation. This aspect and the qualitative findings limit the generalisation of the results and do not allow the authors to find differences among AYCs in terms of specific variables, such as gender. Thus, a larger sample, with subjects recruited from the whole of the country and a wider variety of open-ended questions pertaining to all aspects of AYCs’ daily lives, would be advisable for future research in this area. Moreover, the questionnaire did not include open-ended questions on the reasons why AYCs provide such support or specific questions focused on the positive and negative outcomes of caring for a grandparent; both these aspects should be included in future research to enrich the knowledge on this topic.

Second, this analysis is based on a simultaneous mixed-method design [43], which comprises a qualitatively-driven core component and a quantitative supplementary component. The core and supplemental components were conducted simultaneously so that the quantitative data are not related to the qualitative ones since the latter are solely used for presenting the characteristics of the participants. Further studies based on a sequential study design in which the qualitatively-driven core component and the quantitative supplemental one are conducted sequentially are welcome in order to carry out a more complex investigation of the AYCs’ conditions. Such studies could help shed light on the socioeconomic contextual characteristics within which AYCs live. 

Finally, this study does not consider all the dimensions included in the CSA model, such as the quality of the relationship with the care recipient, mentioned as an “internal mediator factor” in the model. As highlighted in the results section, two respondents stated that their willingness to take on an elder caregiving role is due to love bonds. The link between willingness to care and emotional bonds are key elements of the caregiving relationship mentioned in an earlier study by Dellmann–Jenkins and Brittain [52]. Nevertheless, the qualitative data gathered by the online open-ended questions did not allow the authors to explore this topic. Thus, further qualitative studies able to deepen an understanding of the quality of the intergenerational caregiving relationship and its effect on care-related stress within the framework of the CSA model are welcomed.

Despite these limitations, this study provides new knowledge about AYCs of GrPs in Italy, a country with a low level of awareness, visibility and support of informal caregivers and especially of AYCs. Second, this study provides advances for the formulation of a conceptual theory on AYCs of GrPs starting from a comparison of the core qualitative study findings with the dimensions of the CSA model of Yates [27]. Nevertheless, this has to be considered as a first attempt that needs to be validated and potentially further delineated in future research.

## 5. Conclusions

Drawing on qualitative data from a European online survey on AYCs, this study provides a first conceptual understanding of informal intergenerational caring provided by Italian AYCs of GrPs with reference to the CSA model [27]. The study also provides directions for policymakers and service providers to improve support services targeted at AYCs and the caregiving relationship between AYCs and GrPS in Italy and in countries with similar LTC systems. 

Intergenerational caregiving is itself a complex experience whose effects and meanings include an appraisal process that can prove challenging for AYCs due to the developmental transition from adolescence to adulthood that they are experiencing. Thus, appropriate training and emotional support, partly focused on intergenerational communication and caregiving, could help AYCs of GrPs reflect on their own caregiving role, prevent them from experiencing or at least minimising any negative caregiving outcomes, enhance maturity and empathy and improve the quality of the care relationship.

## Figures and Tables

**Table 1:** Sample characteristics.

Variables	Italian AYCs of GrPs *n* = 87
**Gender**	
Male	27 (31.0%)
Female	58 (66.7%)
Transgender/non-binary	2 (2.3%)
**Age**	
15	10 (11.5%)
16	35 (40.2%)
17	42 (48.3%)
**Country of birth**	
National	81 (93.1%)
Abroad	6 (6.9%)
**Cohabitant**	17 (19.5%)
**At what age did you first begin caring?**	12.8 ± 2.5 *
**What type of health-related condition does the person(s) have:**	
Physical disability	54 (62.1%)
Mental illness	18 (20.7%)
Cognitive impairment	31 (35.6%)
Addiction	7 (8.1%)
Other	14 (16.1%)
**Health problems**	28 (32.2%)
**Formal support services received by AYCs’ family**	20 (23.0%)
**Informal help**	28 (33.3%)
**Hours a day spent providing care**	2.7 ± 2.6 *
**PANOC-YC20** **Positive**	13.9 ± 4.2 *
**PANOC-YC20** **Negative**	2.6 ± 3.2 *
**KIDSCREEN**	34.7 ± 6.7 *

* Note. Data are mean ± sd.

## Data Availability

In accordance with the ME-WE project’s Data Management plan, data collected by means of open-ended “ad-hoc” questions will not be shared. The decision that these data cannot be made publicly accessible is based both on legal and contractual restrictions. Namely, an increased possibility of identifying individual participants through their answers.

## References

[1] Teipel K. (2013). Understanding Adolescence Seeing through a Developmental Lens.

[2] D’Amen B., Socci M., Santini S. (2021). Intergenerational caring: A systematic literature review on young and young adult caregivers of older people. BMC Geriatr..

[3] Boumans N.P., Dorant E. (2018). A cross-sectional study on experiences of young adult carers compared to young adult noncarers: Parentification, coping and resilience. Scand. J. Caring Sci..

[4] Eurostat (2019). Ageing Europe—Looking at the Lives of Older People in the EU. https://ec.europa.eu/eurostat/en/web/products-statistical-books/-/ks-02-19-681.

[5] OECD (2020). Who Cares? Attracting and Retaining Care Workers for the Elderly. https://www.oecd.org/publications/who-cares-attracting-and-retaining-elderly-care-workers-92c0ef68-en.htm.

[6] Me-We Project Consortium (2019). Enabling Young Carers to Pursue Their Goals in Life and Reach Their Full Potential: Converting Research Findings into Policy Actions. https://carers.org/resources/all-resources/107-enabling-young-carers-to-pursue-their-goals-in-life-and-reach-their-full-potential-converting-research-findings-into-policy-actions.

[7] Istat (2015). Anziani: Le Condizioni di Salute in Italia e Nell’unione Europea. https://www.istat.it/it/files//2017/09/Condizioni_Salute_anziani_anno_2015.pdf.

[8] Joseph S., Sempik J., Leu A., Becker S. (2020). Young carers research, practice and policy: An overview and critical perspective on possible future directions. Adolesc. Res. Rev..

[9] Aldridge J. (2018). Where are we now? Twenty-five years of research, policy, and practice on young carers. Crit. Soc. Policy.

[10] Leu A., Becker S. (2014). Young Carers. Obo in Childhood Studies.

[11] Brimblecombe N., Pickard L., King D., Knapp M. (2017). Perceptions of unmet needs for community social care services in E ngland. A comparison of working carers and the people they care for. Health Soc. Care Community.

[12] Rutherford A.C., Bu F. (2018). Issues with the measurement of informal care in social surveys: Evidence from the English Longitudinal Study of Ageing. Ageing Soc..

[13] Nap H.H., Hoefman R., De Jong N., Lovink L., Glimmerveen L., Lewis F., Santini S., D’Amen B., Socci M., Boccaletti L. (2020). The awareness, visibility and support for young carers across Europe: A Delphi study. BMC Health Serv. Res..

[14] Becker S. (2007). Global perspectives on children’s unpaid caregiving in the family: Research and policy on ‘young carers’ in the UK, Australia, the USA and Sub-Saharan Africa. Glob. Soc. Policy.

[15] Dilworth-Anderson P., Williams S.W., Cooper T. (1999). Family caregivers to elderly African Americans: Caregiver types and structures. J. Gerontol. B Psychol. Sci. Soc. Sci..

[16] Katz S. (1983). Assessing Self-maintenance: Activities of Daily Living, Mobility, and Instrumental Activities of Daily Living. J. Am. Geriatr. Soc..

[17] Orel N.A., Dupuy P. (2002). Grandchildren as auxiliary caregivers for grandparents with cognitive and/or physical limitations: Coping strategies and ramifications. Child Study J..

[18] Svanberg E., Stott J., Spector A. (2010). ‘Just helping’: Children living with a parent with young onset dementia. Aging Ment. Health.

[19] Fives A., Kennan D., Canavan J., Brady B. (2013). Why we still need the term ‘young carer’: Findings from an exploratory study of young carers in Ireland. Crit. Soc. Work.

[20] Stamatopoulos V. (2018). The young carer penalty: Exploring the costs of caregiving among a sample of Canadian youth. Child Youth Serv..

[21] Fruhauf C.A., Jarrott S.E., Allen K.R. (2006). Grandchildren’s perceptions of caring for grandparents. J. Fam. Issues.

[22] Carers Trust (2016). Invisible and in Distress: Prioritising the Mental Health of England’s Young Carers. https://carers.org/resources/all-resources/82-invisible-and-in-distress-prioritising-the-mental-health-of-englands-young-carers.

[23] Cohen D., Greene J.A., Toyinbo P.A., Siskowski C.T. (2012). Impact of family caregiving by youth on their psychological well-being: A latent trait analysis. J. Behav. Health Serv. Res..

[24] Doran T., Drever F., Whitehead M. (2003). Health of young and elderly informal carers: Analysis of UK census data. BMJ.

[25] Orel N.A., Dupuy P., Wright J. (2004). Auxiliary caregivers: The perceptions of grandchildren within multigenerational caregiving environments. J. Intergener. Relatsh..

[26] Bolas H., Wersch A.V., Flynn D. (2007). The well-being of young people who care for a dependent relative: An interpretative phenomenological analysis. Psychol. Health.

[27] Yates M.E., Tennstedt S., Chang B.H. (1999). Contributors to and mediators of psychological well-being for informal caregivers. J. Gerontol. B Psychol. Sci. Soc. Sci..

[28] Eurostat (2020). Elderly Population across EU Regions. https://ec.europa.eu/eurostat/web/products-eurostat-news/-/DDN-20200402-1#:~:text=In%202019%2C%2020.3%25%20of%20the,share%20from%20a%20decade%20earlier.

[29] European Commission and Social Protection Committee (2021). 2021 Long-Term Care Report. Trends, Challenges and Opportunities in an Ageing Society.

[30] León M., Pavolini E. (2014). Social Investment’ or Back to ‘Familism’: The Impact of the Economic Crisis on Family and Care Policies in Italy and Spain. South Eur. Soc. Polit..

[31] Courbage C., Montoliu-Montes G., Wagner J. (2020). The effect of long-term care public benefits and insurance on informal care from outside the household: Empirical evidence from Italy and Spain. Eur. J. Health Econ..

[32] Triantafillou J., Naiditch M., Repkova K., Stiehr K., Carretero S., Emilsson T., Di Santo P., Bednarik R., Brichtova L., Ceruzzi F. (2010). Informal Care in the Long-Term Care System. European Overview Paper. http://citeseerx.ist.psu.edu/viewdoc/download?doi=10.1.1.572.1803&rep=rep1&type=pdf.

[33] Verbakel E., Metzelthin S.F., Kempen G.I. (2018). Caregiving to older adults: Determinants of informal caregivers’ subjective well-being and formal and informal support as alleviating conditions. J. Gerontol. B Psychol. Sci. Soc. Sci..

[34] Metzing-Blau S., Schnepp W. (2008). Young carers in Germany: To live on as normal as possible—A grounded theory study. BMC Nurs..

[35] Leu A., Becker S. (2017). A cross-national and comparative classification of in-country awareness and policy responses to “young carers”. J. Youth Stud..

[36] Senato della Repubblica Italiana, XVIII Legislatura Fascicolo Iter DDL S. 1461. Disposizioni per il Riconoscimento ed il Sostegno del Caregiver Familiare. https://www.senato.it/leg/18/BGT/Schede/FascicoloSchedeDDL/ebook/52186.pdf.

[37] Leu A., Guggiari E., Phelps D., Magnusson L., Nap H.H., Hoefman R., Lewis F., Santini S., Socci M., Boccaletti L. (2021). Cross-national Analysis of Legislation, Policy and Service Frameworks for Adolescent Young Carers in Europe. J. Youth Stud..

[38] Barbabella F., Checcucci P., Aversa M.L., Scarpetti G., Fefè R., Socci M., Di Matteo C., Cela E., Damiano G., Villa M. (2020). Le Politiche per L’invecchiamento Attivo in Italia. Rapporto Sullo Stato Dell’arte. http://famiglia.governo.it/media/2132/le-politiche-per-l-invecchiamento-attivo-in-italia.pdf.

[39] Joseph S., Becker S., Becker F., Regel S. (2009). Assessment of caring and its effects in young people: Development of the Multidimensional Assessment of Caring Activities Checklist (MACA-YC18) and the Positive and Negative Outcomes of Caring Questionnaire (PANOC-YC20) for young carers. Child Care Health Dev..

[40] Ravens-Sieberer U., Erhart M., Rajmil L., Herdman M., Auquier P., Bruil J., Power M., Duer W., Abel T., Czemy L. (2010). Reliability, construct and criterion validity of the KIDSCREEN-10 score: A short measure for children and adolescents’ well-being and health-related quality of life. Qual. Life Res..

[41] Ravens-Sieberer U., Herdman M., Devine J., Otto C., Bullinger M., Rose M., Klasen F. (2014). The European KIDSCREEN approach to measure quality of life and well-being in children: Development, current application, and future advances. Qual. Life Res..

[42] Santini S., Socci M., D’Amen B., Di Rosa M., Casu G., Hlebec V., Lewis F., Leu A., Hoefman R., Brolin R. (2020). Positive and Negative Impacts of Caring among Adolescents Caring for Grandparents. Results from an Online Survey in Six European Countries and Implications for Future Research, Policy and Practice. Int. J. Environ. Res. Public Health.

[43] Morse J.M., Niehaus L. (2016). Mixed Method Design: Principles and Procedures.

[44] Strauss A.L., Corbin J.M. (1998). Basics of Qualitative Research: Techniques and Procedures for Developing Grounded Theory.

[45] Novak J.D. (2001). L’apprendimento Significativo.

[46] Pearlin L.I., Mullan J.T., Semple S.J., Skaff M.M. (1990). Caregiving and the stress process: An overview of concepts and their measures. Gerontologist.

[47] Lawton M.P., Kleban M.H., Moss M., Rovine M., Glicksman A. (1989). Measuring caregiving appraisal. J. Gerontol..

[48] Lawton M.P., Moss M., Kleban M.H., Glicksman A., Rovine M. (1991). A two-factor model of caregiving appraisal and psychological well-being. J. Gerontol..

[49] Regulation G.D.P. (2016). Regulation (EU) 2016/679 of the European Parliament and of the Council of 27 April 2016 on the protection of natural persons with regard to the processing of personal data and on the free movement of such data, and repealing Directive 95/46/EC (General Data Protection Regulation). Off. J. Eur. Union.

[50] Barbabella F., Poli A., Chiatti C., Pelliccia L., Pesaresi F., NNA Network Non Autosufficienza (2017). The compass of NNA: The state of the art based on data. Care of Non Self-Sufficient Older People in Italy, 6th Report, 2017–2018.

[51] Fruhauf C.A., Orel N.A. (2008). Developmental issues of grandchildren who provide care to grandparents. Int. J. Aging Hum. Dev..

[52] Dellmann-Jenkins M., Brittain L. (2003). Young Adults’ Attitudes Toward Filial Responsibility and Actual Assistance to Elderly Family Members. J. Appl. Gerontol..

[53] Rith-Najarian L.R., McLaughlin K.A., Sheridan M.A., Nock M.K. (2014). The biopsychosocial model of stress in adolescence: Self-awareness of performance versus stress reactivity. Stress.

[54] Weil L.G., Fleming S.M., Dumontheil I., Kilford E.J., Weil R.S., Rees G., Dolan R.J., Blakemore S.J. (2013). The development of metacognitive ability in adolescence. Conscious. Cogn..

